# Cetuximab Combined With Sonodynamic Therapy Achieves Dual-Modal Image Monitoring for the Treatment of EGFR-Sensitive Non-Small-Cell Lung Cancer

**DOI:** 10.3389/fonc.2022.756489

**Published:** 2022-02-14

**Authors:** Guanhua Qiu, Lianfang Xue, Xiaoqi Zhu, Xiuxin Lu, Lidong Liu, Zhonghai Wang, Xiangdong Li, Cuiqing Huang, Junjie Liu

**Affiliations:** ^1^ Department of Ultrasound and Department of Radiology, Affiliated Tumor Hospital of Guangxi Medical University, Nanning, China; ^2^ Department of Pharmacy, The First Affiliated Hospital of Jinan University, Guangzhou, China; ^3^ Department of Guangxi Medical University, Affiliated Cancer Hospital, Nanning, China; ^4^ Department of Oncology, Jinzhou Central Hospital, Jinzhou, China; ^5^ The Guangzhou Key Laboratory of Molecular and Functional Imaging for Clinical Translation, Jinan University, Guangzhou, China

**Keywords:** NSCLC, EGFR, Cetuximab, nanomaterials, MRI, IR780

## Abstract

**Background:**

Blocking signaling by epidermal growth factor receptor (EGFR), can effectively inhibit the proliferation and differentiation of non-small-cell lung cancer (NSCLC). Additionally, an increasing number of NSCLC patients have treatment limitations caused by EGFR overexpression or mutations. Therefore, we constructed a nanotherapy platform consisting of cetuximab (CTX) to target EGFR-sensitive NSCLC with an iron tetroxide core loading the sound-sensitive agent IR780 for dual-mode imaging diagnosis by combining targeting and sonodynamic therapy (SDT) to reshape the tumor microenvironment (TME), enhance the SDT antitumor effects and improve the therapeutic effects of EGFR sensitivity.

**Methods:**

IR780@INPs were prepared by reverse rotary evaporation, CTX was adsorbed/coupled to obtain IR780@INPs-CTX, and the morphology and structure were characterized. Intracellular ROS levels and cell apoptosis first verified its killing effects against tumor cells. Then, a nude mouse lung cancer subcutaneous xenograft model was established with HCC827 cells. A real-time fluorescence IVIS imaging system determined the targeting and live distribution of IR780@INPs-CTX in the transplanted tumors and the imaging effects of the T2 sequence of the INPs by magnetic resonance imaging (MRI) 0 h, 2 h, 4 h and 6 h after administration to confirm drug efficacy.

**Results:**

*In vitro*, US+IR780@INPs-CTX produced a large amount of ROS after SDT to induce cell apoptosis, and significant cell death after live/dead staining was observed. *In vivo* fluorescence imaging showed the IR780@INPs-CTX was mainly concentrated in the tumor with a small amount in the liver. MRI displayed rapid enrichment of the IR780@INPs into tumor tissue 0h after injection and the T2 signal intensity gradually decreases with time without obvious drug enrichment in the surrounding tissues. *In vivo*, at the end of treatment, the US+IR780@INPs-CTX group showed disappearance or a continued decrease in tumor volume, indicating strong SDT killing effects.

**Conclusion:**

The combination of CTX and SDT is expected to become a novel treatment for EGFR-sensitive NSCLC.

## Introduction

Lung cancer is a major public health problem worldwide and the leading cancer-related cause of death. The number of lung cancer patients and deaths due to lung cancer are increasing yearly (1–3). In 2020, according to GLOBOCAN statistics, there were 2.21 million new lung cancer cases (accounting for 11.4% of the total number of cancer cases) and 1.8 million deaths (accounting for 18% of the total number of cancer deaths) (4). Among them, non-small-cell lung cancer (NSCLC) is the main histological subtype of this disease (85%) (5). Due to the lack of typical clinical manifestations and diagnostic methods for early stage lung cancer, most NSCLC patients are diagnosed at advanced stages (6), and their 5-year survival rate is as low as 5%-15% (7). The current treatments for lung cancer are mainly chemotherapy, radiotherapy and surgery (8).

Epidermal growth factor receptor (EGFR) is a transmembrane tyrosine kinase receptor, and its signaling pathway plays a vital role in cell cycle progression and physiological and biochemical processes (9, 10). Therefore, EGFR overexpression or mutation is closely related to tumors, especially NSCLC (11). Notably, the proportion of Asian patients with EGFR mutations is as high as 30%-40% (12). In recent years, EGFR has been shown to be an important target in the treatment of NSCLC. A growing number of patients develop inherent drug resistance caused by EGFR mutations (13), which seriously affects their clinical prognosis (14–16). Therefore, there is an urgent need for new combined treatment methods to accurately enhance the early diagnosis rate of NSCLC while also effectively improving the limitations of targeted therapy and tumor cell resistance. To this end, we herein propose a new treatment model to improve the efficiency of clinical treatment in response to the above problems.

**Scheme 1:**
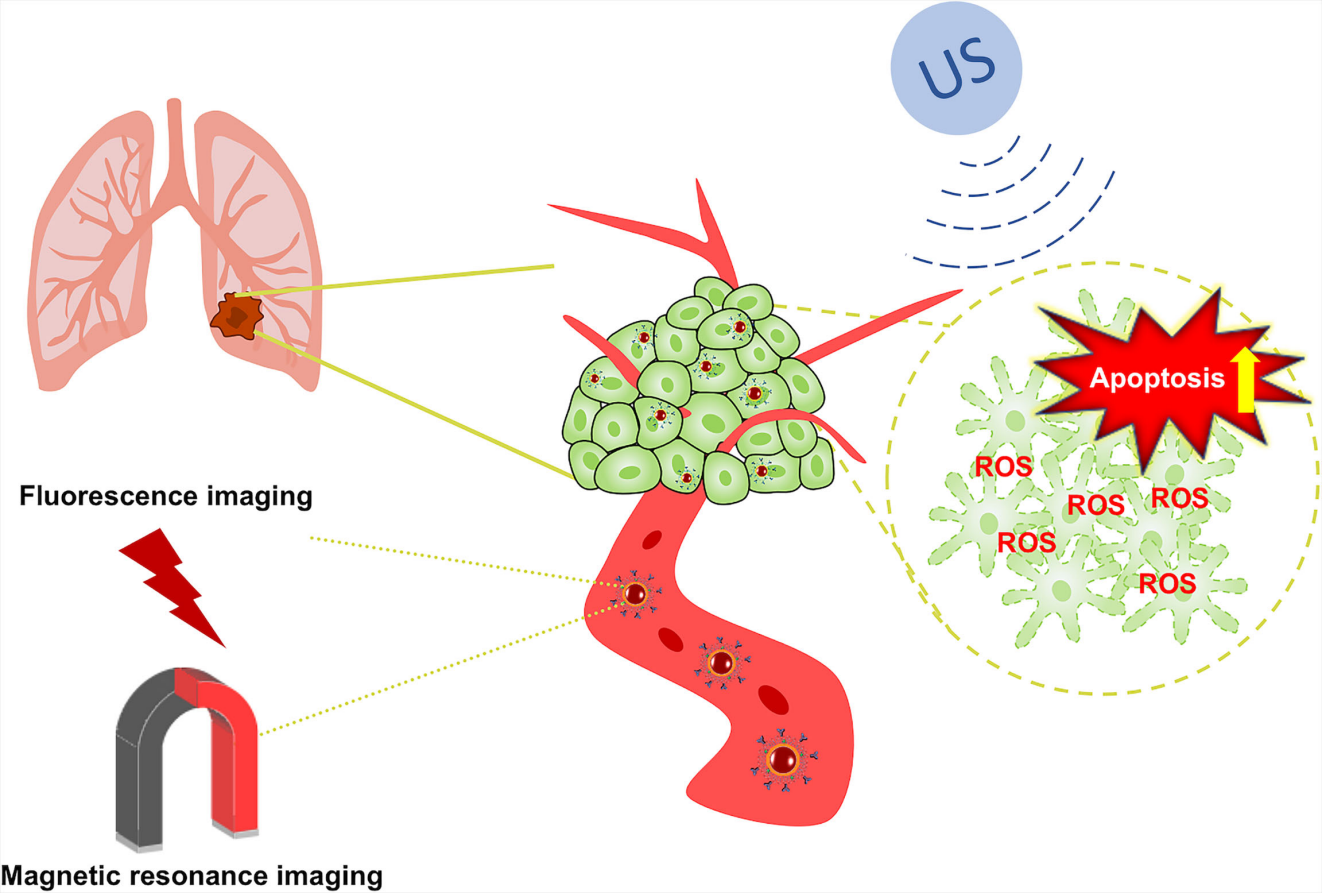
Schematic illustration of the process of US+IR780@INPs-CTX for cancer therapy.

In recent years, photodynamic therapy (PDT), photothermal therapy (PTT), and sonodynamic therapy (SDT) have gradually been noticed by scientific researchers. Compared with PDT and PTT, SDT is noninvasive, highly targeted, inexpensive and easy to perform. These advantages of SDT have added a whole new dimension to the clinical treatment of tumors (17). SDT synergistically combines ultrasound with sonosensitizers. It uses ultrasound to penetrate deep tissues and focuses on specific areas to activate the sonosensitizers that are enriched in the tumor tissues, finally promoting the production of ROS to induce cell apoptosis (18, 19). The cavitation effects produced also reshape the tumor microenvironment (TME). The application of nanomaterials for clinical treatment modalities has undergone revolutionary changes through its advantages of high specific surface area, drug transport and release, and improved biocompatibility (20), but problems continue to exist that limit their therapeutic effects, such as poor chemical stability, high toxicity, and poor targeting. As an essential element for the human body, iron plays a central role in the process of DNA replication and is also an auxiliary factor for heme (21, 22). Moreover, the application of iron-containing contrast agents in magnetic resonance imaging (MRI) has gradually matured due to the advantages of their good imaging effects, low toxicity, and low price (23, 24). On the other hand, a large number of studies have shown that IR780 can be effectively imaged after high-intensity infrared radiation to mediate PTT, PDT and SDT in tumor tissue (25). Therefore, we prepared a Fe_3_O_4_ nanoparticle carrier (INPs), loaded the sonosensitizer IR780 inside the INPs (IR780@INPs), and coupled the macromolecular monoclonal antibody cetuximab (IR780@INPs-CTX) to inhibit EGFR, and achieve bidirectional induction of apoptosis. in turn induces apoptosis (**Scheme 1**).

## Materials and Methods

### Preparation of the Nanoparticles

First, 100 mg of DSPE-PEG-COOH, 10 mg of IR780 and 10 mg of Fe3O4 were dissolved in 4 mL of chloroform and mixed well with ultrasound treatment. Reverse rotary evaporation at 70°C and 20 rpm was then performed and pure water was added to obtain the IR780@INPs.

IR780@INPs (20 mg) were dispersed in MES solution. Additionally, 20 mg of CTX (5 mg/mL) was added for adsorption at 37°C for 1 h. 1-Ethyl-3-(3-dimethylaminopropyl)carbodiimide (EDC) was added to 15 mM MES to make a 10 mg/mL solution, which coupled overnight at 37°C, followed by centrifugal ultrafiltration and purification with pure water. The final IR780@INPs-CTX were stored in PBS.

### Characterization of the Nanoparticles

The morphology and structure of the INPs nanomaterials were characterized by transmission electron microscopy (TEM; JEOL-2100F, Japan). A Zetasizer Nano ZS90 from Malvern was used to analyze the particle size and potential distribution of the material. Vibrating sample magnetometer (VSM; Quantum Design, USA) was used to evaluate the superparamagnetism of the INPs, and the absorption spectrum was obtained with an ultraviolet spectrophotometer (UV3600 Shimadzu, Japan). The measurement range was 300-1000 nm with a step length of 1 nm. To measure the absorbance of the drug, we used a microplate reader to measure the absorbance at the characteristic wavelength of the drug at concentrations of 1, 2, 2.5, and 5 μg/mL. The absorbance was plotted as a function of drug concentration to construct a standard curve, and from this curve, the packaging rate and drug loading could be determined. To observe the interaction between the drug and the blood, we collected 2 mL of whole blood from mouse eyeballs into an anticoagulation tube, centrifuged the sample, and removed the supernatant to obtain the red blood cells. The red blood cells were then washed with PBS 3 times and resuspended in PBS. Next, prepared solutions of drugs at different concentrations were added to the red blood cell suspensions followed by incubation in a 37°C hypoxic incubator for 6 h or 12 h. Finally, each sample was centrifuged and the OD value of the supernatant was measured at 540 nm with a microplate reader.

### Cell Line and Culture

HCC827, A549 cells were purchased from the American Type Culture Collection (Manassas, VA, USA). The HCC827 cells were cultured in RPMI 1640 medium (Gibco, USA) and A549 cells were cultured in DMEM (Gibco, USA), and 10% fetal bovine serum (FBS; Gibco, USA), 100 U/mL penicillin and 100 μg/mL streptomycin (Solarbio, China) were added at 37°C under 5% CO2 (Beijing).

### Cellular Uptake

To prove the targeting properties of the drug and its phagocytosis by cells, HCC827 (mutant EGFR) cells and A549 (wild-type EGFR) cells were inoculated into a specific laser confocal culture dish and incubated overnight in a hypoxic incubator. Then, IR780@INPs and IR780@INPs-CTX were administered separately, and incubation continued for 1 h after the corresponding drug treatment. Subsequently, the supernatant was aspirated and the cells were washed with PBS 3 times. The nuclei were stained with 80 µl Hoechst 33342 for 15 min and the cells were washed 3 times with PBS. Finally, confocal laser scanning microscopy (CLSM; Leica SP8, Germany) was used to image and observe the cells. The excitation wavelength was 750 nm and the emission wavelength was 820 nm.

### Detection of ROS Generation

The fluorescent probe 2’,7’-dichlorofluorescein diacetate (DCFH-DA) was used to detect the level of intracellular ROS. We established five groups: the control group, IR780@INPs group, IR780@INPs-CTX group, US+IR780@INPs group and US+IR780@INPs-CTX group. HCC827 cells (5×10^6^ cells per well) were seeded in a 6-well plate (Corning, NY, USA) and placed in a hypoxic incubator for 24 h. Cells in the logarithmic growth phase were treated with the appropriate drug or control reagents followed by incubation for 1 h. Next, the DCFH-DA fluorescent probe was diluted to an appropriate volume and added to the cell culture medium in the dark for the immediate performance of SDT. The ultrasonic irradiation parameters were as follows: power density = 1 W/cm^2^, transducer frequency = 1 MHz, duty cycle = 50, and duration = 3 min. The agar gel was placed between the orifice plate and the US probe. Then, an inverted fluorescence microscope was used to observe ROS staining in the cells, and images were acquired for processing.

### Cell Viability and Apoptosis

Calcein-AM and Propidium Iodide (PI) can detect cell endoplasmic enzyme activity and cell membrane integrity, respectively, thereby reflecting cell activity and cytotoxicity. To understand the killing effects of the drugs on cells more intuitively, HCC827 cells (5×10^6^ cells per well) were seeded in a 6-well plate and cultured in a hypoxic incubator for 24 h. Cells in the logarithmic growth phase were treated with the appropriate drug followed by incubation for 24 h. The cells were then stained with both Calcein-AM and PI in the dark. Finally, the staining of dead and live cells was observed with an inverted fluorescence microscope and images were acquired for processing.

### Animal Model

SPF-grade 4-week-old female nude mice (Experimental Animal Center of Guangxi Medical University), weighing 12~15 g, were bred in the SPF-grade Animal Experimental Center. HCC827 cells (2×10^6^) were resuspended in 200 μl of PBS and subcutaneously injected into the left armpits of the nude mice to establish a mouse lung cancer subcutaneous xenograft model. All animal experiments were approved by the Ethics Committee of the Affiliated Tumor Hospital of Guangxi Medical University.

### Fluorescence Imaging *In Vivo*

Fluorescence imaging was used to study the enrichment of the targeted drug (IR780@INPs-CTX) in mice. When the mouse tumor diameters approached 5 mm, injected 100 µl of IR780@INPs or IR780@INPs-CTX through the tail vein respectively. Then, at different times (0 h, 2 h, 4 h, and 6 h after injection), a Xenogen IVIS spectral imaging system coupled with a Cool CCD camera (Xenogen Company, USA) in combination with an 808 nm fluorescent laser (power = 1.5 W, 850/50 filter) was used to scan the mice at an excitation wavelength of 750 nm and an emission wavelength of 820 nm. After 24 h of imaging, the mice were sacrificed by cervical dislocation, and the tumors and major organs were excised for further fluorescence imaging.

### MRI *In Vivo*

We used a 3.0T MRI imaging system (GE, USA) to obtain T2-weighted MRI images through multilayer multiecho sequences. The scanning conditions were as follows: 16×16 cm^2^ field of view, 2.0 mm layer thickness, 4 excitation times, and an image size of 256×256. The repetition time was 180 ms, and the echo time was 6.0 ms.

For analysis of the drug images of the nude mice from the MRI scans, the mouse tumor diameters were close to 5 mm. Then, we injected 100µl of IR780@INPs or IR780@INPs-CTX through the tail veins of the mice respectively, and T2-weighted images were obtained on a 3.0T MRI system at different times (0 h, 2 h, 4 h and 6 h after injection) to evaluate the targeting properties of IR780@INPs and IR780@INPs-CTX to mouse tumors and obtain good imaging results.

### Anticancer Therapy *In Vivo*

The tumor-bearing mice were randomly divided into 5 groups: the control group, IR780@INPs group, IR780@INPs-CTX group, US+IR780@INPs group and US+IR780@INPs-CTX group. When the tumors grew to approximately 50 mm^3^, the tumor-bearing mice in each experimental group were treated *via* the tail vein with the appropriate drug a total of three times with a one day interval between each treatment. Additionally, the US+IR780@INPs and US+IR780@INPs-CTX groups were administered SDT for 6 h. During the experiment, the body weights, body temperatures and other health conditions of the mice were monitored every 3 days, and the tumor sizes were measured with a caliper. The standard formula:


$$V=lenggth*width*\frac{width}{2}$$

was used to calculate the tumor volumes. 21 days after treatment, the mice were sacrificed by cervical dislocation, the main organs (heart, liver, spleen, lung and kidney) and the tumor were analyzed by immunohistochemical fluorescence, and serological indicators (UA, ALT, TP, CysC, UREA, ALB II, CCR, AST) were evaluated to determine the effects of the drugs on liver and kidney function during treatment.

### Immunofluorescence

The tumors and main organs of the mice sacrificed after treatment were excised for pathological analysis, including Ki67, α-SMA, HIF-1α, EGFR and TUNEL immunofluorescent staining. The excised tumor tissues were fixed with 4% paraformaldehyde and embedded in paraffin. The tissue was cut into 5 µm thick sections and deparaffinized, followed by staining. Slides were mounted after staining, and finally, all sections were observed and images were recorded under a microscope.

### Statistics

Each experiment was repeated at least 3 times. Data are expressed as the mean ± SD. The paired 1-tailed Student’s t test was used to examine differences between the means of two groups. A P value of less than 0.05 was considered a statistically significant difference.

## Results

### Characterization of Nanoparticles

**Figure 1:**
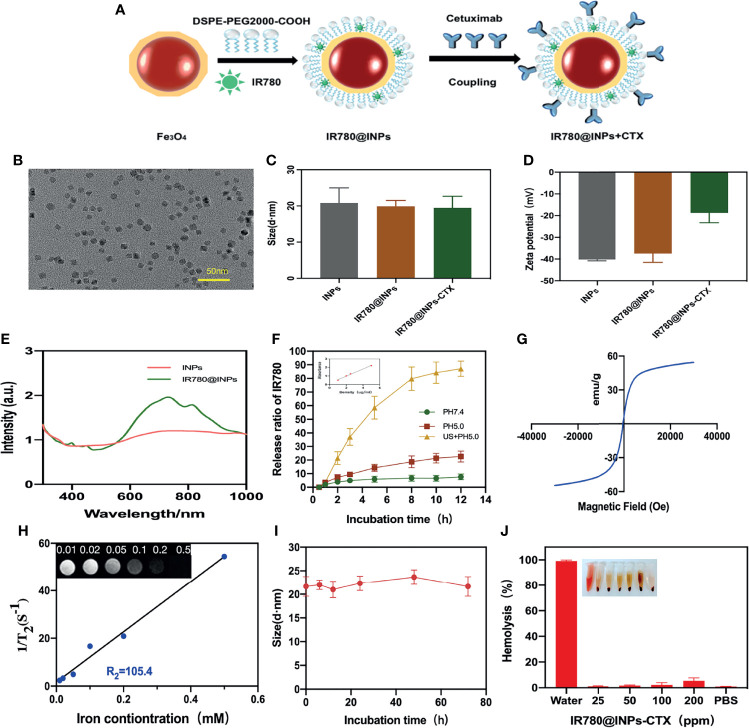
**(A)** Preparation process of IR780@INPs-CTX. **(B)** TEM images of INPs. **(C, D)** DLS and Zeta detection results of INPs loaded with IR780 and CTX. **(E)** Uv-vis spectrum of IR780@INPs. **(F)**The release curve of IR780 under different conditions of pH 7.4, pH 5.0 and US + pH 5.0, and the inset is the image of standard curve of IR780. **(G)** Magnetization curve of IR780@INPs. **(H)**T2 relaxation rate of IR780@INPs nanoparticles, and the inset is T2-weighted images of IR780@INPs nanoparticles under different iron concentrations (mM). **(I)** Stability curves of IR780@INPs-CTX in PBS for 0 h, 6 h, 12h, 24h, 48h and 72h. **(J)** Stability evaluation of IR780@INPs-CTX in serum.

**Figure 1A** demonstrates the synthesis process of nanomaterial INPs, and the PEG-modified nanoparticles have good dispersibility, the particle size distribution is uniform, and there is no observable aggregation (**Figure 1B**), From **Figure 1C**, it can be seen that the particle size of the nanoparticles in aqueous solution is approximately 20 nm, **Figure 1D** shows that the zeta potential range of the INPs is negative. From the results of the potential range of the targeted group and the nontargeted group, the surface potentials of CTX and the carboxylated INPs are significantly reduced, confirming that CTX was coupled to the surface of the INPs. According to **Figure 1E**, the characteristic absorption peak of the IR780@INPs is at approximately 760 nm. From the standard curve in the inset of **Figure 1F**, the encapsulation rate of IR780 was 61.2%, and the drug loading rate was 2.74%. Ultrasound can significantly stimulate IR780 release (**Figure 1F**). **Figure 1G** displays good superparamagnetism of the INPs, and **Figure 1H** shows that an R² value of 101.8, as measured by MRI T2-weighted imaging. **Figures 1I, J** demonstrates that IR780@INPs-CTX maintain good solution stability and compatibility *in vitro*.

### Cellular Uptake

**Figure 2:**
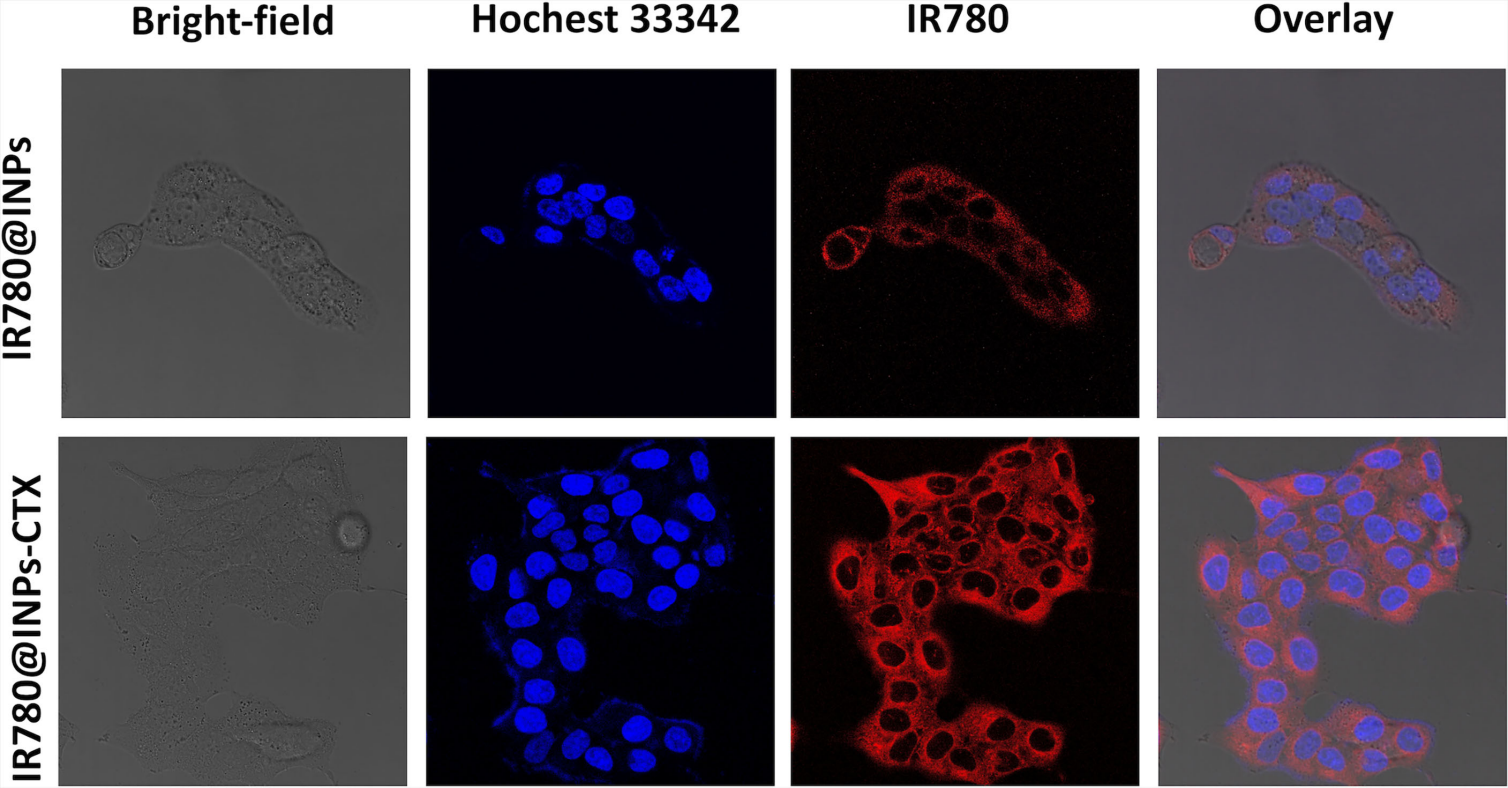
Confocal microscopic images of HCC827 cells incubated with IR780@INPs and IR780@INPs-CTX (red). Cell nucleus were dyed with Hochest333442 (blue).

Drug phagocytosis by HCC827 cells and A549 cells was analyzed by CLSM. **Figures 2**, **S1** show that in the group of A549 cells, the nucleoli were uniformly blue-stained, the red fluorescence around the nucleus was extremely weak. The results of phagocytosis of HCC827 cells showed that the nucleoli of the two groups of cells (treated with IR780@INPs-CTX and IR780@INPs) were evenly stained blue. In the IR780@INPs-CTX group, there was a strong red color surrounding the nucleus, indicating that IR780@INPs-CTX were effectively phagocytosed by HCC827 cells. The phagocytosis of IR780@INPs-CTX in the targeted group by the HCC827 cells was significantly higher than that of the nontargeted IR780@INPs and the negative target control group A549, proving that HCC827 cells had greater uptake of the targeted drug through the EGFR receptor-mediated pathway.

### Detection of ROS Generation

**Figure 3:**
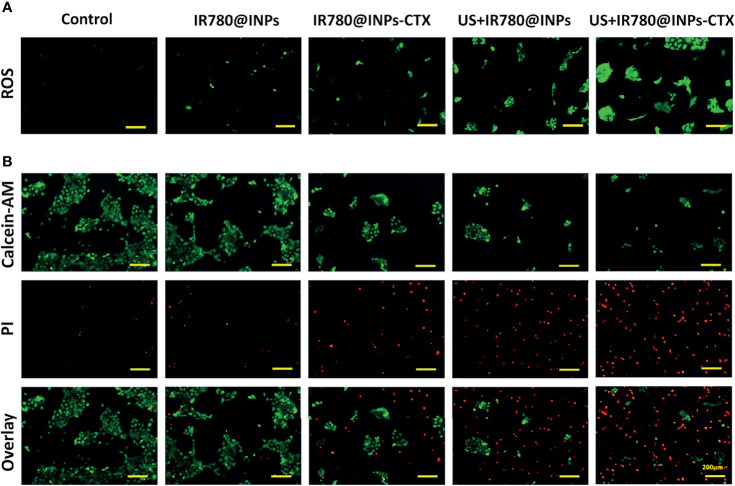
**(A)** ROS levels of HCC827 cells in the five groups. **(B)** In different groups, each group of cells is stained with Calcein-AM and PI, and the living cells are green fluorescent, and dead cells are red fluorescent.

From the ROS detection results (**Figures 3A**, **S2**), only a slight green fluorescence signal was observed in the cells in the IR780@INPs and IR780@INPs-CTX groups, which were not been irradiated by ultrasound, indicating that these cells produce a very small amount of ROS. Additionally, there was almost no green fluorescence signal in the untreated control group. Interestingly, strong green fluorescence signals were found in the cells in the US+IR780@INPs and US+IR780@INPs-CTX groups treated with ultrasound, and the intracellular signal in the targeted US+IR780@INPs-CTX group was stronger, again showing that SDT can further promote the production of ROS.

### Cell Viability and Apoptosis

To prove that SDT can effectively enhance the lethality of the drug in HCC827 cells, we used a more detailed method. The live/dead cell staining experiment (**Figure 3B**) showed that most HCC827 cells were viable after treatment with IR780@INPs-CTX, displaying a wide range of green fluorescence signals. However, cells treated with US+IR780@INPs-CTX exhibited strong red fluorescence signals, and there were very few surviving cells in the field of view. This shows that SDT can cause a large amount of tumor cell necrosis.

### *In Vivo* Imaging Assay

**Figure 4:**
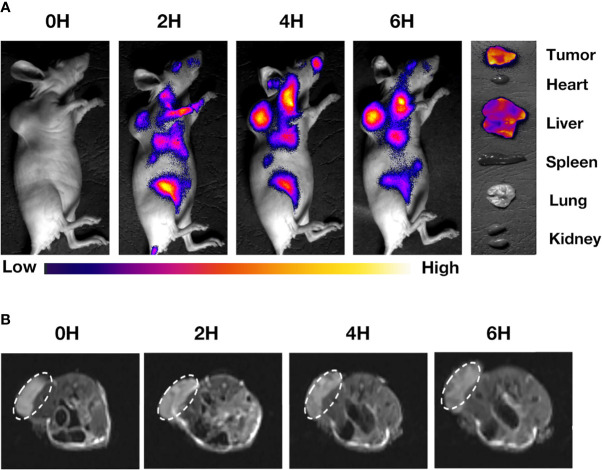
**(A)** The distribution of drugs in tumors and main organs of mice after 0 h, 2 h, 4 h, and 6 h after injection of IR780@INPs-CTX *via* tail vein. **(B)** MRI T2-weighted imaging of mice in IR780@INPs-CTX groups at 0 h, 2 h, 4 h and 6 h after drug injection.

A real-time fluorescent IVIS imaging system was used to analyze tumor-specific targeting and the live distribution of IR780@INPs-CTX in nude mice with subcutaneously transplanted tumors. **Figures 4A**, **S3** displays the concentration of the drug in the tumors of nude mice at 0 h, 2 h, 4 h and 6 h after tail vein injection of IR780@INPs or IR780@INPs-CTX. Fluorescence imaging shows that there is no strong fluorescence signal, and there is no obvious difference from the surrounding organs of the tumor-bearing mice in IR780@INPs group, However the mice in IR780@INPs-CTX group, the drug quickly accumulated in the tumor after administration, the fluorescence signal was significantly higher in the tumor than in the surrounding tissues, the fluorescent signal gradually increased over time, and the fluorescence signal in the main organs gradually weakened. This result verified that IR780@INPs-CTX strongly targets EGFR on HCC827 cells and has good fluorescence imaging effects. Then, the nude mice were sacrificed, and the tumors and major organs were dissected and analyzed by fluorescence imaging with the IVIS imaging system. The results showed that the IR780@INPs-CTX clearly targeted the tumor and concentrated there, while a small amount collected in the liver. The drug was found in the liver because that is their site of metabolism.

To prove the imaging effects of the T2 sequence of the INPs for magnetic resonance, we injected the drug into mice through the tail vein and observed them *via in vivo* MRI. **Figures 4B**, **S4** display that the IR780@INPs was rapidly enriched in the tumor tissue from 0 h after injection. T2-weighted imaging showed that the signal intensity decreased gradually with time, and there was no obvious drug found in the surrounding tissues. But in IR780@INPs group, T2-weighted imaging showed that the signal intensity did not change significantly.

### Anticancer Therapy *In Vivo*

**Figure 5:**
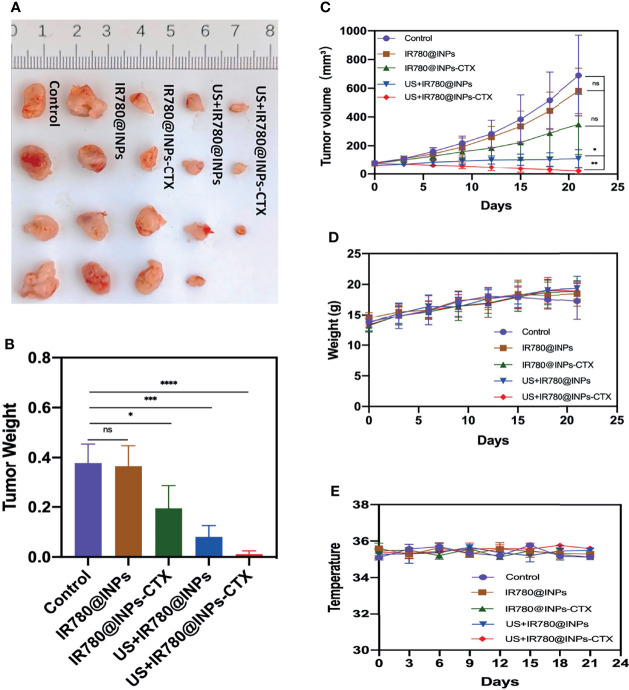
**(A)** Anatomical image of tumors in different groups of mice after 21 days of treatment. **(B)** Weight of isolated tumors in different treatment groups after 21 days of therapy. **(C)** Volume of isolated tumors after 21 days of therapy. **(D)** Changes in body weight of mice during treatment. **(E)** Changes in mice body temperature during treatment. ns, P > 0.05; *P < 0.05; **P < 0.01; ***P < 0.001; ****P < 0.0001.

**Figure 6:**
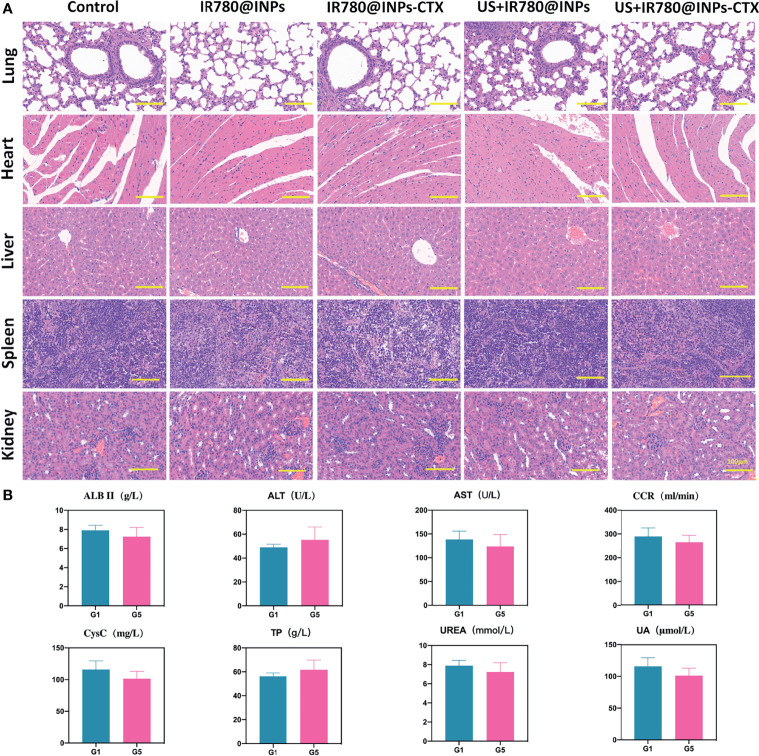
*In vivo* toxicity evaluation of IR780@INPs-CTX. **(A)** H&E stained pictures of the main organs (lung, heart, liver, spleen and kidney) of mice, the scale bar is 100μm. **(B)** Serological indicator examination of tumor-bearing mice in each group after treatment (G1: Control, G5: US+IR780@INPs-CTX), p<0.05 shows no significant difference between G1 and G5.

To verify the antitumor effects of SDT combined with the INPs containing CTX *in vivo*, we constructed a lung cancer model under the skins of nude mice to analyze the therapeutic effects of each treatment on the tumors. As shown in **Figure 5A**, the targeted US+IR780@INPs-CTX group had the strongest inhibitory effect on tumor growth, and the sizes of the mouse tumors were significantly reduced or even disappeared. It is worth noting that although the US+IR780@INPs group had a slightly enhanced inhibitory effect on tumor growth compared with the IR780@INPs-CTX group, its effect was less than that of the targeted US+IR780@INPs-CTX group. The tumor changes in each experimental group also proved the good drug transport abilities of INPs *in vivo*. This result also shows that CTX can effectively deliver nanoparticles into tumor tissues, thereby inhibiting the proliferation of tumor cells and enhancing the killing effect through ultrasound treatment and sonosensitizers. **Figures 5B–E** show that the tumor weights and tumor growth curve trends *in vivo* were consistent, and the weights and body temperatures of the mice did not change significantly. At the same time, through the H&E staining results of the main organs and the statistical results of serological indicator (**Figures 6A, B**), it can be seen that within the 21 days, the drugs of each group have no obvious toxic effect on the mice, which ensures the biological safety.

### Immunofluorescence

**Figure 7:**
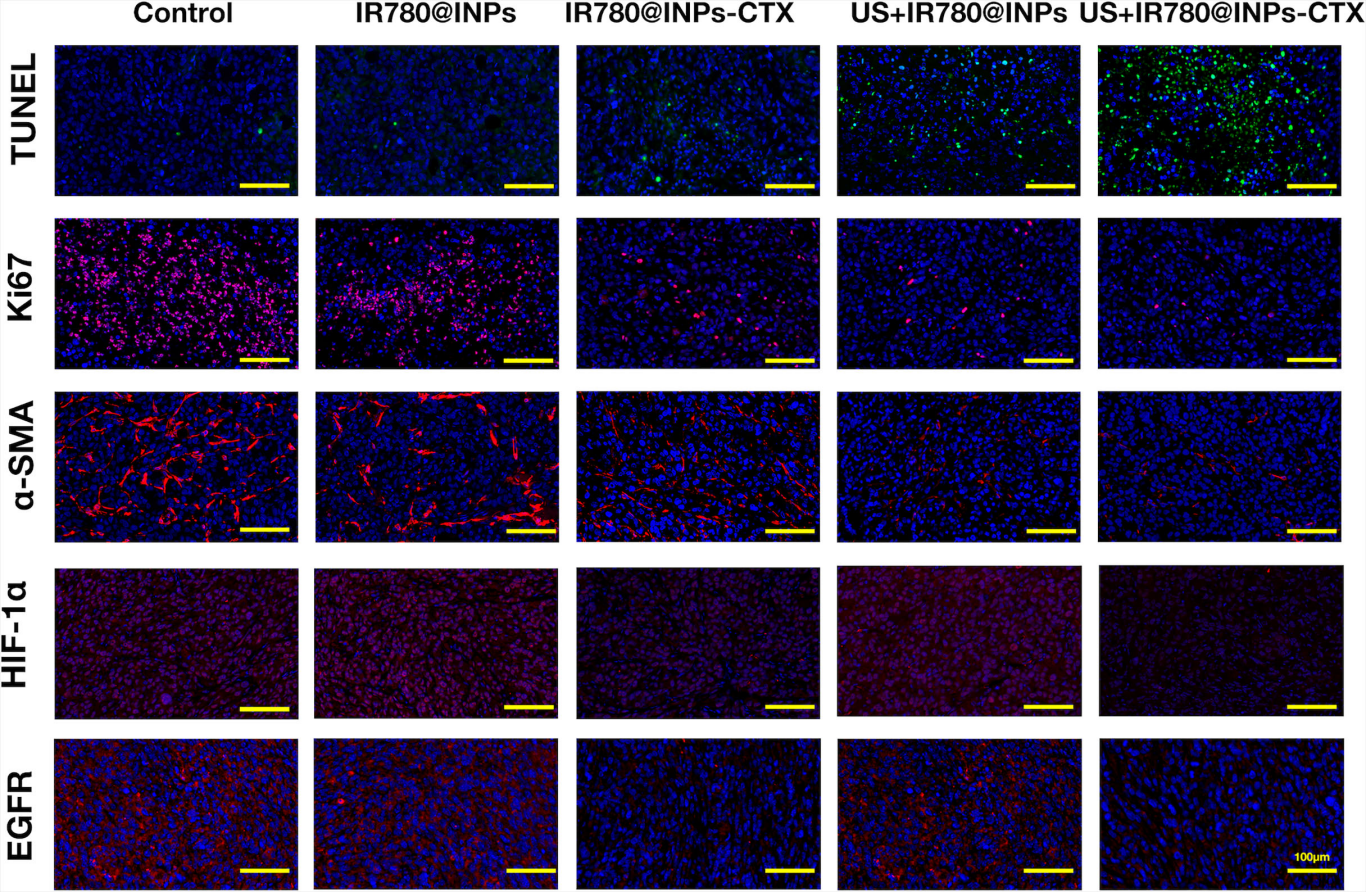
Ki-67, α-SMA, EGFR, HIF-1α and TUNEL immunofluorescence staining detection pictures of tumor sections in each group (the scale bar is 200μm).

It can be seen from the Ki67 results in **Figures 7**, **S5** that few red fluorescence signals were detected in the blue-stained nuclei in the US+IR780@INPs-CTX group, followed by the US+IR780@INPs group and the IR780@INPs-CTX group. The fluorescence in the IR780@INPs group was not very different from that in the control group. Comparing each treatment group, the tumor proliferation of the targeted US+IR780@INPs-CTX group was extremely low, so it was assumed that this treatment had great inhibitory effects on the proliferation of tumor tissue. TUNEL and Ki67 staining showed the opposite trend. As an indicator of tumor fibrosis, α-SMA represents the degree of tumor malignancy. The ratio of α-SMA in the US+IR780@INPs and US+IR780@INPs-CTX groups showed a significant decreasing trend. The HIF-1α results suggested that IR780@INPs-CTX and US+IR780@INPs-CTX reduced the HIF-1α expression level, while in the US+IR780@INPs group, HIF-1α expression slight increased compared to the control group. The EGFR results showed that the expression of EGFR was decreased in the IR780@INPs-CTX and US+IR780@INPs-CTX groups, which is precisely because CTX can specifically combine with EGFR, thereby competitively blocking the expression of EGFR.

## Discussion

In this experiment, we developed US+IR780@INPs-CTX nanomaterials to achieve a targeted therapy platform for NSCLC dual-modal imaging diagnosis and synergistic sonodynamic therapy.

Compared with traditional nanomaterials, our iron nanoparticles have a particle size of less than 20 nm. In theory, loading more drug may affect the particle size of nanomaterials (26). After IR780 and CTX were loaded into the INPs, the particle size did not obviously change, which indicated that the particle size of IR780@INPs-CTX can be controlled in a stable and uniform range; this is also more conducive to the drug reaching TME (27). Moreover, to overcome the poor tumor selectivity of traditional nanoparticles (28), we carried the monoclonal antibody CTX on the surface of the INPs, which can effectively recognize EGFR (29). This can kill tumor cells and also serve as a target for SDT to further enhance drug enrichment in tumor tissue and improve the efficiency of targeted therapy (30, 31). We have herein shown through *in vivo* and *in vitro* experiments that IR780@INPs-CTX can achieve maximum targeted accumulation in tumor cells under the guidance of CTX. These results indicate that therapeutic drugs can be delivered to tumor tissues more quickly, optimizing the therapeutic effect and further reflecting the good development prospects of molecular targeted imaging technology.

As a noninvasive treatment method, SDT has good tumor killing effects (32, 33). We used the targeting effects of CTX to cause a large amount of internalization of the sonosensitizer IR780 into tumor cells. Additionally, ultrasound has been used to locate deep tissue cells and activate tumor tissue. The accumulated sonosensitizer can kill tumor cells, which is mainly achieved by the generation of ROS after SDT. High levels of ROS can further promote DNA double-strand breaks, thereby destroying DNA and inducing cell apoptosis (34, 35). Comparison of the IR780@INPs-CTX group and the US+IR780@INPs-CTX group *in vitro* from the ROS detection and the live/dead cell staining experiments, the IR780@INPs-CTX group without US treatment produced only a small amount of ROS, and the number of dead cells after drug treatment was also very small. However, the US+IR780@INPs-CTX group showed a large amount of ROS production the increase in the number of dead cells was very obvious. Our experimental mechanism is consistent between the *in vivo* and *in vitro* experiments. *In vivo*, we observed changes in the tumor sizes in each group. Interestingly, during the initial stage of treatment, the difference between the US+IR780@INPs-CTX and US+IR780@INPs groups was not significant. At the end of the treatment period, the tumor volume in the US+IR780@INPs-CTX group continued to decrease or the tumors disappeared. These results show that SDT has a strong killing effect on tumor tissues, but it remains difficult to achieve a thorough treatment effect alone. Notably, although the tumor volumes of the mice the US+IR780@INPs group decreased slowly in the later stage, it showed a significant improvement compared with the control group. Therefore, we used the combination treatment of the monoclonal antibody CTX along with SDT to compensate for the CTX resistance mutation that occurs after the long-term treatment of NSCLC (36). ROS can significantly promote tumor cell apoptosis and inhibit proliferation, while SDT can effectively decrease the content of collagen fibers, thereby increasing drug infiltration at the tumor site. Surprisingly, the IR780@INPs-CTX group, and especially the US+IR780@INPs-CTX group, showed a decrease in HIF-1α level. In the complex TME, hypoxia causes HIF-1α to be overexpressed. SDT is a highly oxygen-consuming process. After a single US+IR780@INPs SDT treatment, the expression of HIF-1α increased slightly. In contrast, HIF-1α levels in the IR780@INPs-CTX and US+IR780@INPs-CTX groups decreased. The main reason for this result may be that tumor cells often have abnormal glucose metabolism, and uncontrolled amplification patterns are frequently accompanied by upregulation of EGFR expression. EGFR participates in the regulation of oxidative stress and further downregulates HIF-1α, thereby promoting ROS generation and expanding the therapeutic benefits.

The experiments performed here show that the targeted nanodiagnostic agent US+IR780@INPs-CTX exhibits good imaging results, combining highly sensitive fluorescence imaging with high spatial resolution MRI, and this continuous treatment system more effective against NSCLC with high EGFR expression, providing new ideas for future clinical diagnosis and treatment programs.

## Data Availability Statement

The raw data supporting the conclusions of this article will be made available by the authors, without undue reservation.

## Ethics Statement

The animal study was reviewed and approved by Affiliated Tumor Hospital of Guangxi Medical University.

## Author Contributions

JL, XDL, and CH designed the study and revised the manuscript. LX participated in designing and conducting the research. GQ conceived the research and contributed to the writing of the manuscript. XZ and XXL collected and analyzed the data. LL and ZW interpreted the results. All authors have read and approved the manuscript.

## Funding

This work was supported by the National Natural Science Foundation of China (82160341), the Natural Science Foundation of Guangxi Province (2017GXNSFAA198260) and Guangxi Key R&D Program (AB18221089).

## Conflict of Interest

The authors declare that the research was conducted in the absence of any commercial or financial relationships that could be construed as a potential conflict of interest.

## Publisher’s Note

All claims expressed in this article are solely those of the authors and do not necessarily represent those of their affiliated organizations, or those of the publisher, the editors and the reviewers. Any product that may be evaluated in this article, or claim that may be made by its manufacturer, is not guaranteed or endorsed by the publisher.
