# Supplementary material for: Cetuximab Combined With Sonodynamic Therapy Achieves Dual-Modal Image Monitoring for the Treatment of EGFR-Sensitive Non-Small-Cell Lung Cancer
Source: Front Oncol. 2022 Feb 14;12:756489. doi: 10.3389/fonc.2022.756489 (PMC8886674; doi:10.3389/fonc.2022.756489)
Supplement: Supplementary file 1 [file DataSheet_1.docx]

Supplementary Material

# Supplementary Data


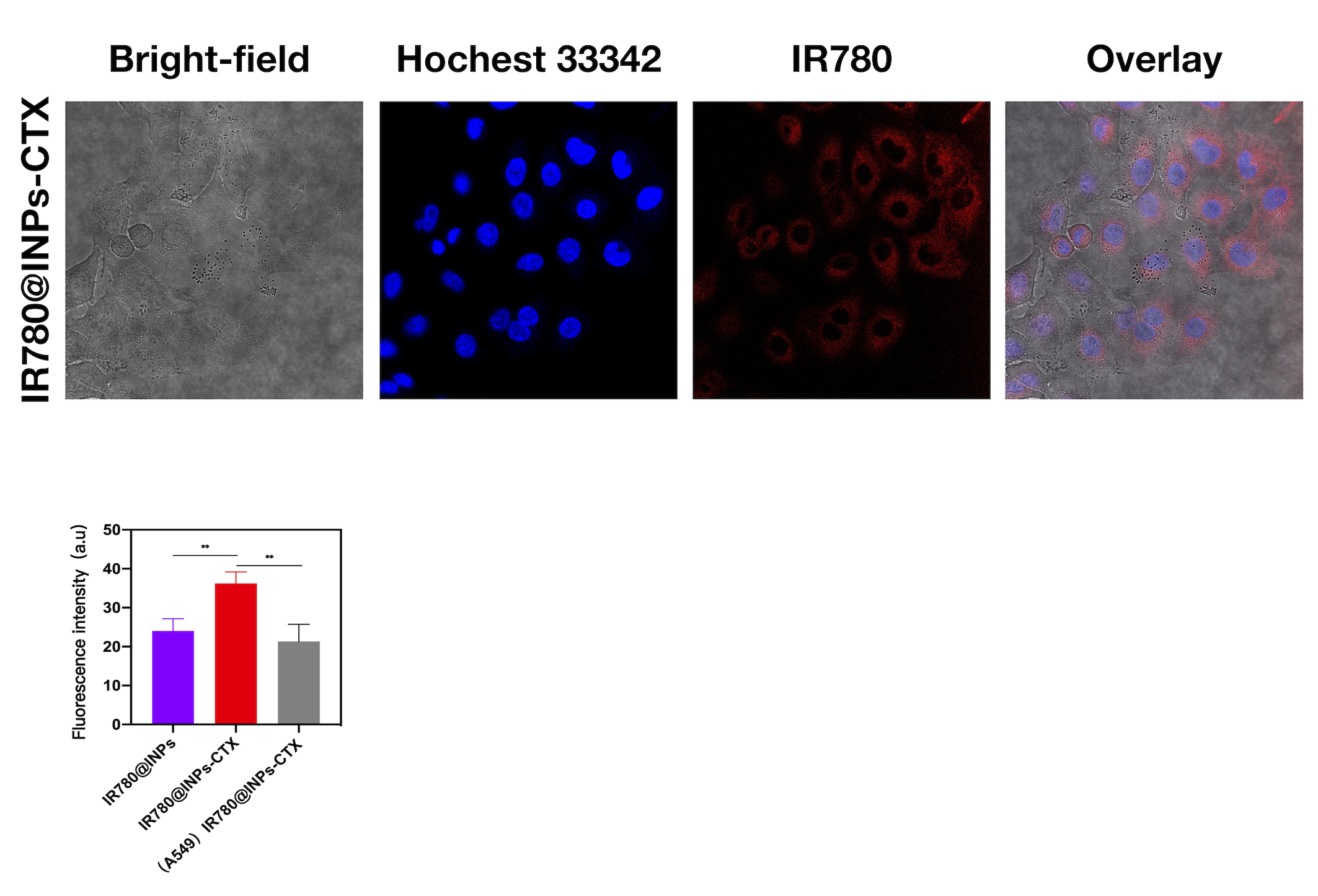


**Figure S1：**Confocal microscopic images of A549 cells incubated with IR780@INPs-CTX (red). Cell nucleus were dyed with Hochest333442 (blue)；quantitative analysis of fluorescence intensity. (**p < 0.01).


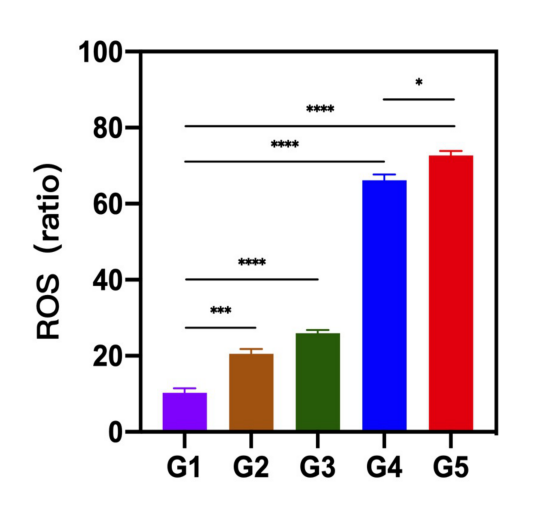


**Figure S2：**Quantitative analysis of ROS generation. (G1: Control, G2: IR780@INPs, G3: IR780@INPs-CTX, G4: US+IR780@INPs, G5: US+IR780@INPs-CTX)(****p<0.0001,***p < 0.001, **p < 0.01, or *p < 0.05).


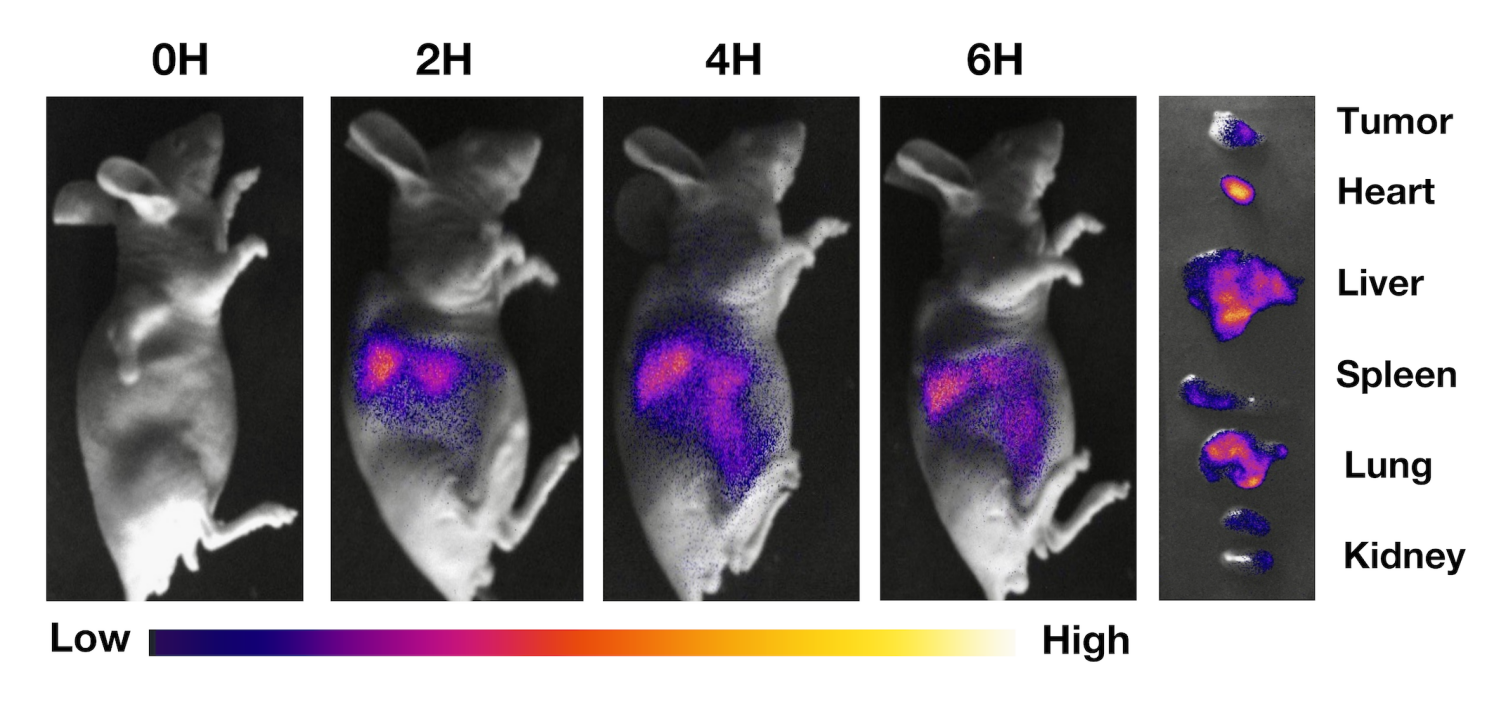


**Figure S3：**The distribution of drugs in tumors and main organs of mice after 0 h, 2 h, 4 h, and 6 h after injection of IR780@INPs via tail vein.


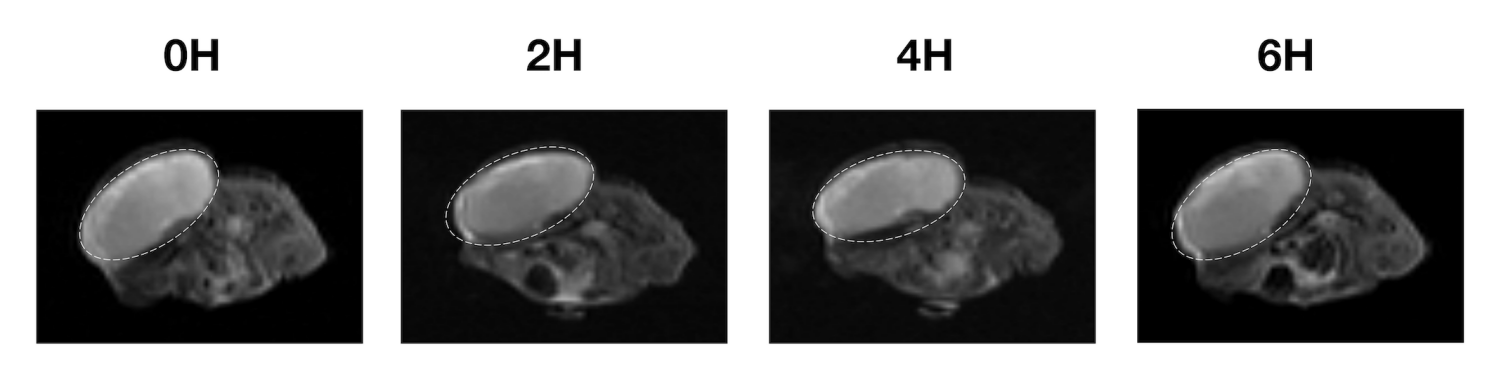


**Figure S4：**MRI T2-weighted imaging of mice in IR780@INPs groups at 0h、2h、4h and 6h after drug injection.


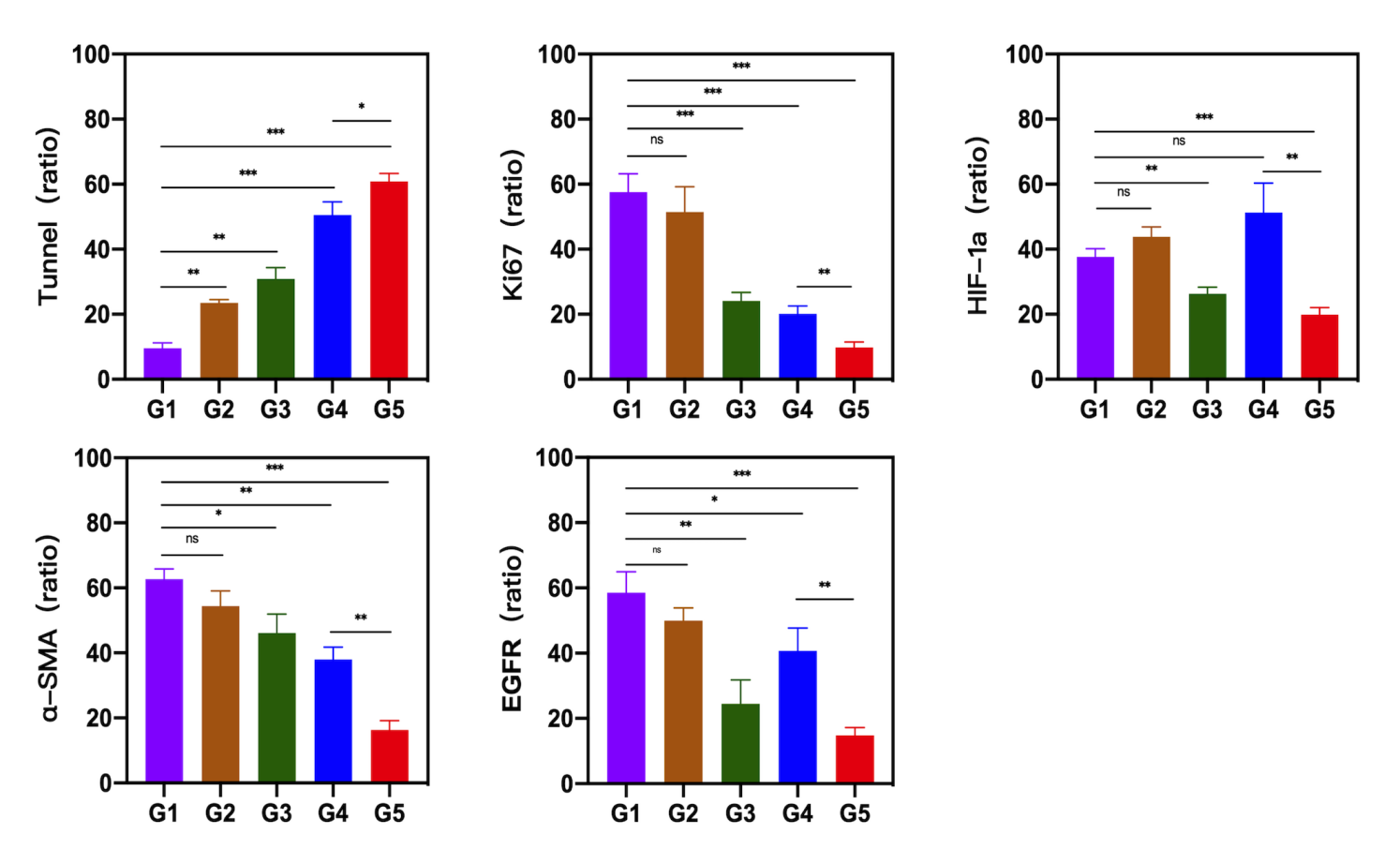


**Figure S5：**Quantitative analysis of fluorescence intensity. (G1: Control, G2: IR780@INPs, G3: IR780@INPs-CTX, G4: US+IR780@INPs, G5: US+IR780@INPs-CTX)(****p<0.0001,***p < 0.001, **p < 0.01, or *p < 0.05).
