# Supplementary material for: Cetuximab Combined With Sonodynamic Therapy Achieves Dual-Modal Image Monitoring for the Treatment of EGFR-Sensitive Non-Small-Cell Lung Cancer
Source: Front Oncol. 2022 Feb 14;12:756489. doi: 10.3389/fonc.2022.756489 (PMC8886674; doi:10.3389/fonc.2022.756489)
Supplement: Supplementary file 2 [file DataSheet_2.docx]

Original file upload link ：

https://www.jianguoyun.com/p/DdmCy4YQ_JbfCRj-s4YE
